# Omicron SARS-CoV-2 maternal infection is associated with favorable fetomaternal outcomes and fewer placental vascular malperfusion lesions compared with previous variants

**DOI:** 10.1016/j.xagr.2026.100690

**Published:** 2026-08-30

**Authors:** Annabelle Remoué, Yurina Suazo, Claire de Moreuil, Karine Morcel, Christophe Tremouilhac, Camille Sauvée, Arnaud Uguen, Pascale Marcorelles

**Affiliations:** 1 Service d’anatomie et cytologie pathologiques, CHU de Brest, Brest, France (Remoué, Suazo, Uguen, and Marcorelles); 2 GETBO (groupe d’Etude de la Thrombose de Bretagne Occidentale), Inserm UMR 1304, Université de Brest, CHU de Brest, Brest, France (Remoué and Moreuil); 3 Service de médecine interne, CHU de Brest, Brest, France (Moreuil); 4 Service de gynécologie-obstétrique, CHU de Brest, Brest, France (Morcel); 5 Service de gynécologie-obstétrique, CH des Pays de Morlaix, Morlaix, France (Tremouilhac); 6 Service de gynécologie-obstétrique, CH de Cornouaille, Quimper, France (Sauvée); 7 LBAI (lymphocytes B, autoimmunité et immunothérapies), Inserm UMR 1227, Université de Brest, CHU de Brest, Brest, France (Uguen); 8 LIEN (laboratoire interactions épithéliums neurones), EA 4586, Université de Brest, CHU de Brest, Brest, France (Marcorelles)

**Keywords:** Omicron variant, pathology, placenta, placental inflammation, placental malperfusion, SARS-CoV-2

## Abstract

**BACKGROUND:**

The SARS-CoV-2 pandemic, declared in March 2020, has affected over 570 million people worldwide. The impact on pregnant women and fetuses remains unclear, particularly with the Omicron variant, which appears to increase the infection rates.

**OBJECTIVE:**

This retrospective study describes fetal-maternal complications and placental features in Omicron-variant SARS-CoV-2 infections during pregnancy and compares them with those associated with other variants and with noninfected controls from published studies examining the association between SARS-CoV-2 infection and placental pathology.

**STUDY DESIGN:**

We analyzed all consecutive placentas referred to our hospital from May 2021 to March 2023, associated with proven Omicron-variant SARS-CoV-2 maternal infection during pregnancy.

**RESULTS:**

Among the 315 placentas studied, fetal-maternal complication rates were comparable with those in the general population, including preeclampsia (0.6%), intrauterine growth restriction (5%), small for gestational age (9.1%), and preterm delivery (3.1%). We observed a significantly lower rate of hypotrophic placentas below the 10th percentile (4.1% vs 15.0% [*P*<0.001]), chronic deciduitis (3.8% vs 10.5% [*P*<0.01]), maternal vascular malperfusion (49.2% vs 58.8% [*P*<0.01]), and fetal vascular malperfusion lesions (7.6% vs 14.5% [*P*<0.001]) in the Omicron cohort vs published data from studies of individuals with non–Omicron variant SARS-CoV-2. We did not observe an increased incidence of chronic inflammation or the SARS-CoV-2 placentitis histologic triad in comparison to control cases from published data. A high rate of acute histological chorioamnionitis was observed, without an increased incidence in preterm deliveries. The interval between infection and delivery did not alter placental features.

**CONCLUSION:**

This large Omicron-variant placentas cohort highlighted clinically, an overall good outcome, and histologically significantly less vascular malperfusion and chronic deciduitis than in non–Omicron SARS-CoV-2 variants.

**AJOG Global Reports at a Glance**

**Why was this study conducted**

To explore specifically clinical and histological impact of Omicron SARS-CoV-2 variant on the pregnancy. To describe placental histological patterns considering the delay between the maternal infection and the delivery.

**Key findings**

There is no difference in clinical outcomes in comparison with the general population. There are fewer vascular malperfusion lesions and less chronic deciduitis compared with non–Omicron variant SARS-CoV-2. No cases of SARS-CoV-2 placentitis were identified among the cohort of examined placenta. No difference in placental histopathological findings was observed considering the delay between maternal infection and delivery.

**What does this add to what is known?**

This is the first cohort specifically focusing on placentas with maternal Omicron infection. It demonstrates a favorable clinical prognosis supported by robust histological data showing fewer lesions of vascular malperfusion.

## Introduction

The outbreak of the SARS-CoV-2 virus began in December 2019, and the World Health Organization (WHO) declared it a global pandemic in March 2020.^1^ Since then, the virus has spread extensively, infecting more than 570 million people in 225 countries and resulting in more than 6.3 million confirmed deaths.^2^

Scientific data on the impact of the virus on pregnancy and fetuses are still incomplete, although a teratogenic effect seems to be ruled out. Pregnancy-related maternal and fetal complications, including preeclampsia and preterm delivery, have been reported after the emergence of the δ variant and were more prevalent during the third trimester of pregnancy.^3^^,^^4^ The virus has been shown to induce direct damage to endothelial cells, leading to thrombosis, inflammation, immune response dysregulation, and alterations in pathways related to angiotensin-converting enzyme 2 (ACE2), which could promote the emergence of hypertensive disorders of pregnancy.^5^^,^^6^

The SARS-CoV-2 virus is a highly dynamic pathogen that constantly acquires new mutations, leading to the emergence of various variants. The Omicron variant (B.1.1.529), which emerged in late 2021,^7^ has been noteworthy for its high number of mutations (precisely 50), which have been shown to enhance its ability to evade the immune system.^3^ These mutations are hypothesized to increase the virus’s ability to infect pregnant women by strengthening the interaction between its spike protein and the ACE2 receptor.^5^

A wide variability of macroscopic and histological placental lesions has been described in cases of maternal infection with SARS-CoV-2 during pregnancy, including acute histological chorioamnionitis, chronic villitis, chronic histiocytic intervillositis (CHI), fetal vascular malperfusion (FVM), maternal vascular malperfusion (MVM), placental abruption, massive perivillous fibrin deposition, and intervillous thrombosis.^8^, ^9^, ^10^, ^11^, ^12^, ^13^, ^14^ However, the main limitations of most published studies are the lack of stratification of placental lesions according to the SARS-CoV-2 variant and the absence of an appropriate control group. Within this extensive pathological variability, certain emerging trends have been identified. A characteristic histopathological pattern of SARS-CoV-2 placentitis, consisting of massive perivillous fibrin deposition, intervillositis with numerous neutrophils and trophoblast necrosis (the so-called placentitis triad), has been consistently reported by several independent groups, particularly during the circulation of the δ variant, and has been associated with severe placental injury and adverse fetal outcomes.^15^, ^16^, ^17^, ^18^, ^19^, ^20^, ^21^, ^22^ Only 1 case of SARS-CoV-2 placentitis has been reported in the context of maternal infection with the Omicron SARS-CoV-2 variant; this patient was receiving anti-CD20 immunotherapy.^23^

Some studies have suggested that the timing of SARS-CoV-2 infection could explain the diversity of placental histological lesions reported in the literature, with a 14-day cut-off after maternal infection commonly being adopted.^24^, ^25^, ^26^, ^27^, ^28^, ^29^ This timeframe corresponds to the median duration between symptom onset and viremia clearance.^27^, ^28^, ^29^, ^30^

This study analyzed a retrospective cohort of consecutive placentas from pregnant women infected with the Omicron SARS-CoV-2 variant addressed in our pathology department. Our objective were as follows: (1) to describe the acute and nonacute placental histological patterns associated with Omicron-variant infection during pregnancy and (2) to evaluate the impact of maternal infection on delivery. We compared our results with those reported for other variants in published SARS-CoV-2 placental cohorts.

## Materials and methods

### Case selection

A database query was conducted to identify all placentas from pregnancies affected by maternal SARS-CoV-2 infection that were analyzed at the Pathology department of Brest University Hospital between May 2021 and March 2023. These placentas were referred to our hospital from 6 obstetrics departments (Brest University Hospital, Quimper, Landerneau, and Morlaix General Hospitals) in Brittany, France. A subset of cases underwent placental analysis in accordance with indications validated by scientific guidelines: preterm delivery (10 cases), preeclampsia (2 cases), small for gestational age (SGA) or fetal growth restriction (FGR) (47 cases), term fetal death (1 case), or fetal perinatal complications. Most of the cases underwent placental analysis solely on the grounds of maternal COVID-19 infection during pregnancy.

**Figure 1:**
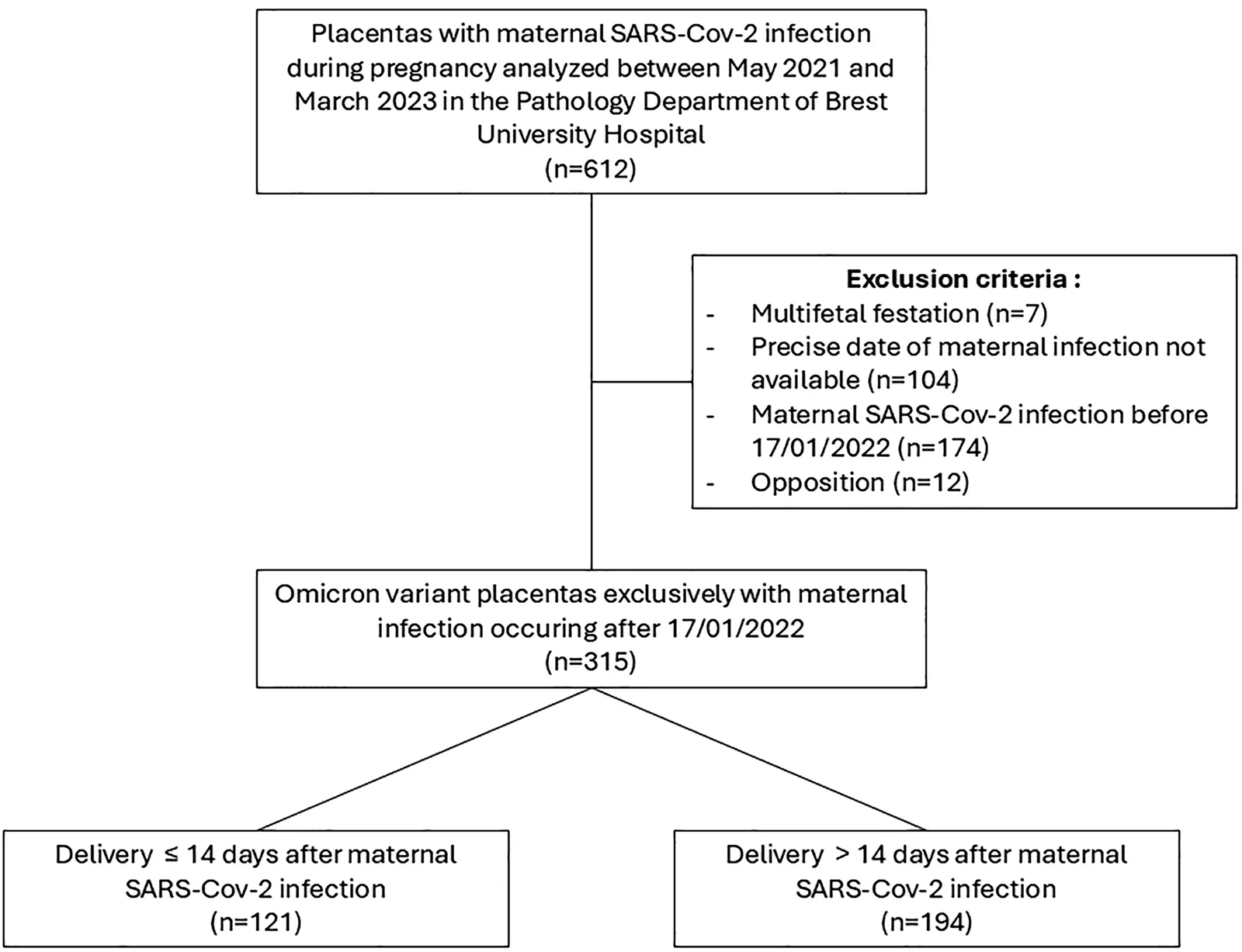
Flow chart of the study A total of 612 placentas were identified from pregnancies with maternal SARS-CoV-2 infection between May 2021 and March 2023; 315 were associated with the Omicron variant after applying exclusion criteria. Two groups were defined based on the interval between maternal infection and delivery.

Omicron was the only circulating variant in Brittany from January 17, 2022, until March 2023, referencing epidemiological data from Santé Publique France^30^; therefore, we excluded cases occurring before that date. All cases for which the date of SARS-CoV-2 infection during pregnancy was unknown were excluded, as were twin pregnancies and cases involving another viral infection. All included cases had a confirmed diagnosis of SARS-CoV-2 by nasopharyngeal polymerase chain reaction test. Of note, 11 women had complete COVID-19 vaccination, and the information was unavailable for the other patients. We do not have information on any prior COVID-19 infection occurring before the pregnancy. A letter of nonopposition was sent to each patient, and patients who refused to participate were excluded, in accordance with the recommendations of the Ethics Committee of the CHU de Brest, protocol 29BRC24.0295. This cohort was separated into 2 groups according to the delay between maternal infection and delivery, with a cutoff of 14 days. Maternal characteristics and fetomaternal complications were collected. The study flowchart is presented in Figure 1.

Remoué. Omicron SARS-CoV-2 infection and placental outcomes. Am J Obstet Gynecol Glob Rep 2026.

### Placental analysis

Placentas were fixed in 4% buffered formalin for 4 days before undergoing macroscopic examination by a senior pathologist. At least 4 samples were collected for each case. These samples were fixed in paraffin and analyzed microscopically using hematoxylin-eosin-saffron staining. Placental lesions were classified according to the Amsterdam consensus classification.^31^

When microscopic evidence of nonacute placental inflammation was observed, immunohistochemistry was performed using the Ventana Benchmark Ultra system (Roche Diagnostics, Meylan, France) and anti-CD3 antibody (polyclonal; 1:100 dilution) (Dako, Santa Clara, CA), anti-CD68 antibody (clone PG-M1; 1:100 dilution) (Dako, Santa Clara, CA), anti-myeloperoxidase antibody (polyclonal; 1:300 dilution) (Dako, Santa Clara, CA), anti-CD20 antibody (clone L26; 1:100 dilution) (Dako, Santa Clara, CA), anti-CD138 antibody (monoclonal, MI15; M7228; 1:50 dilution,) (Dako, Santa Clara, CA), and anti-SARS-CoV-2 N protein antibody (clone AMC0368; 1:100 dilution) (ABclonal, Woburn, MA).

### Comparison with control cohort and previous-variants cohort

To analyze the results observed in our Omicron cohort, a comparison with a control cohort (based on the published data of individuals without SARS-CoV-2 from previous studies examining the association between SARS-CoV-2 infections and placental pathology) and a cohort of previous COVID-19 variants was performed using selected published studies identified after a PubMed search with the keywords COVID-19, SARS-CoV-2, placenta, or pregnancy.^8^^,^^12^^,^^24^^,^^27^^,^^29^^,^^32^, ^33^, ^34^, ^35^, ^36^ As most of the of studies included all variants—α, γ, β, and δ—without distinction, we selected studies that predated November 9, 2021, the date of the discovery of the first Omicron case according to the WHO,^7^ except for Shanes et al,^34^ who specified their results for each variant identified.

### Statistical analysis

Categorical variables were compared using the chi square test or Fisher exact test, as appropriate. Continuous variables were compared using the unpaired Student *t* test or the Mann-Whitney U test, as appropriate. Statistical significance was set at *P*<.05. Analyses were performed using GraphPad Prism version 10.6.1 (GraphPad Software, San Diego, CA).

## Results

A cohort of 315 placentas was constituted, with 121 cases in the acute group, with delivery occurring less than 14 days after the infection, and 194 cases in the nonacute group. In the acute group, the mean interval between maternal infection and delivery was 5.31 days (range, 0–14 days), with a median of 5 days (Q1=1 and Q3=9). In the nonacute group, the mean interval between maternal infection and delivery was 45 days (range, 15–174 days), with a median of 37.5 days (Q1=24 and Q3=62).

The maternal and placental characteristics are summarized in Table 1.

**Table 1:** Fetomaternal characteristics and placental pathologic findings of pregnancies affected by Omicron infection

Characteristics	All population of Omicron infected pregnancies (n=315)	Acute group ≤14 d (n=121)	Non acute group> 14 d (n=194)	*P* value^a^
Clinical characteristics				
Maternal age (y), mean (SD)	18–44 (31)	20–42 (30)	18–44 (31)	.050
Gestational diabetes, n (%)	37 (11.7)	13 (10.7)	24 (12.3)	.720
Preeclampsia, n (%)	2 (0.6)	0 (0)	2 (1.0)	.520
Gestational age at time of Omicron infection, WG (mean)	17–41 (35+3)	29–41 (38+3)	17–39 (33+3)	<.001^a^
Term at delivery (WG)	27–42 (39+3)	31–42 (39+3)	27–42 (39+3)	.710
Premature delivery (<37 WG), n (%)	10 (3.1)	4 (3.3)	6 (3.1)	>.990
Maternal SARS-CoV-2 infection at second trimester (15-28 WG), n (%)	17 (5.3)	0 (0)	17 (8.7)	<.001^a^
Maternal SARS-CoV-2 infection at third trimester (>28 WG), n (%)	298 (94.6)	121 (100)	177 (91.2)	<.001^a^
SGA, n (%)	31 (9.8)	14 (11.5)	17 (8.7)	.440
FGR, n (%)	16 (5)	6 (4.9)	10 (5.1)	>.990
Placental macroscopic findings				
Placental weight <10th percentile, n (%)	13 (4.1)	15 (12.3)	31 (15.9)	.410
Infarct (after 37 WG), n (%)	120 (38.0)	39 (32.2)	81 (41.7)	.090
>5%	13 (4.1)	7 (5.7)	6 (3.0)	.250
Infarct (before <37 WG), n (%)	3 (0.9)	1 (0.8)	2 (1.0)	>.990
Intervillous thrombosis, n (%)	71 (22.5)	23 (19.0)	48 (24.7)	.260
Subchorionic focal thrombosis, n (%)	62 (19.6)	25 (20.6)	37 (19.0)	.770
Placental macroscopic findings				
MVM, n (%)	155 (49.2)	56 (46.2)	99 (51.0)	.420
FVM, n (%)	24 (7.6)	6 (4.9)	18 (9.2)	.190
ACA maternal response, n (%)	174 (55.2)	71 (58.6)	103 (53.0)	.350
Stage 1 Grade 1, n (%)	113 (35.8)	46 (38.0)	67 (34.5)	-
Stage 1 Grade 2, n (%)	37 (11.7)	17 (14.0)	20 (10.3)	-
Stage 2 Grade 1–Grade 2, n (%)	14 (4.4)–10 (3.1)	4 (3.3)–4 (3.3)	10 (5.1)–6 (3.0)	-
ACA fetal response, n (%)	49 (15.5)	21 (17.3)	28 (14.4)	.520
Stage 1 Grade 1–Grade 2, n (%)	39 (12.3)–4 (1.2)	19 (15.7)–1 (0.8)	20 (10.3)–3 (1.5)	-
Stage 2 Grade 1–Grade 2, n (%)	3 (0.9)–3 (0.9)	1 (0.8)–0 (0)	2 (1.0)–3 (1.5)	-
VUE, n (%)	47 (14.7)	17 (14.3)	30 (14.9)	.870
Subchorionic, n (%)	16 (5.0)	9 (7.4)	7 (3.6)	-
Parabasal/paraseptal, n (%)	35 (11.1)	13 (10.7)	22 (11.3)	-
Midparenchyma, n (%)	25 (7.9)	8 (6.6)	17 (8.7)	-
High grade, n (%)	11 (3.4)	4 (3.3)	7 (3.6)	-
Low grade, n (%)	36 (11.4)	13 (10.7)	23 (11.8)	-
CHI low grade, n (%)	6 (1.9)	2 (1.6)	4 (2.0)	>.990
Chronic deciduitis, n (%)	12 (3.8)	6 (4.9)	6 (3.0)	.540

Comparison between acute and nonacute groups.

*ACA*, acute chorioamnionitis; *CHI*, chronic histiocytic intervillitis; *FGR*, fetus growth retardation; *FVM*, fetal vascular malperfusion; *MVM*, maternal vascular malperfusion; *SD*, standard deviation; *SGA*, small for gestational age; *VUE*, villitis of unknown etiology; *WG*, week of gestation.

a *P* value between “acute” and “nonacute” groups.

### Maternal characteristics

Maternal age ranged from 18 to 44 years, with a mean (± standard deviation [SD]) age of 31 (± 5.06) years. A small number of pregnancy-related maternal comorbidities were reported, including gestational diabetes (11.7%) and preeclampsia (0.6%). Of note, 6 cases exhibited antenatal ultrasound anomalies detected during the second trimester of pregnancy before infection, including tetralogy of Fallot, DiGeorge syndrome, fetal cardiac septal hypertrophy, left renal dysplasia, corpus callosum agenesis, and hyperechogenic digestive loops.

SARS-CoV-2 maternal infection occured predominantly during the third trimester (94.6%) of pregnancy. No cases of maternal infection were reported during the first trimester, and only 17 cases (5.3%) occurred during the second trimester. The mean (± SD) gestational age at delivery was 39 (± 1.70) weeks of gestation. Of the 315 cases, 10 (3.1%) pregnancies resulted in preterm deliveries. SGA fetuses were reported in 31 cases (9.1%), and FGR was reported in 16 cases (5%). Only one case of stillbirth occurred at 40 weeks of gestation in a eutrophic fetus (3700 g) after an otherwise uncomplicated pregnancy, apart from the infection at 34 weeks of gestation. Placental examination did not reveal any pathological abnormalities, and no fetal autopsy was performed in accordance with the parents’ wishes.

### Placental Characteristics

Based on the reference values established by Pinar et al,^37^ only 4.1% of the placentas fell below the 10th percentile. Significant macroscopic infarcts were observed in 13 cases (4.1%), including 3 preterm placentas. No cases of excess fibrin were reported.

**Figure 2:**
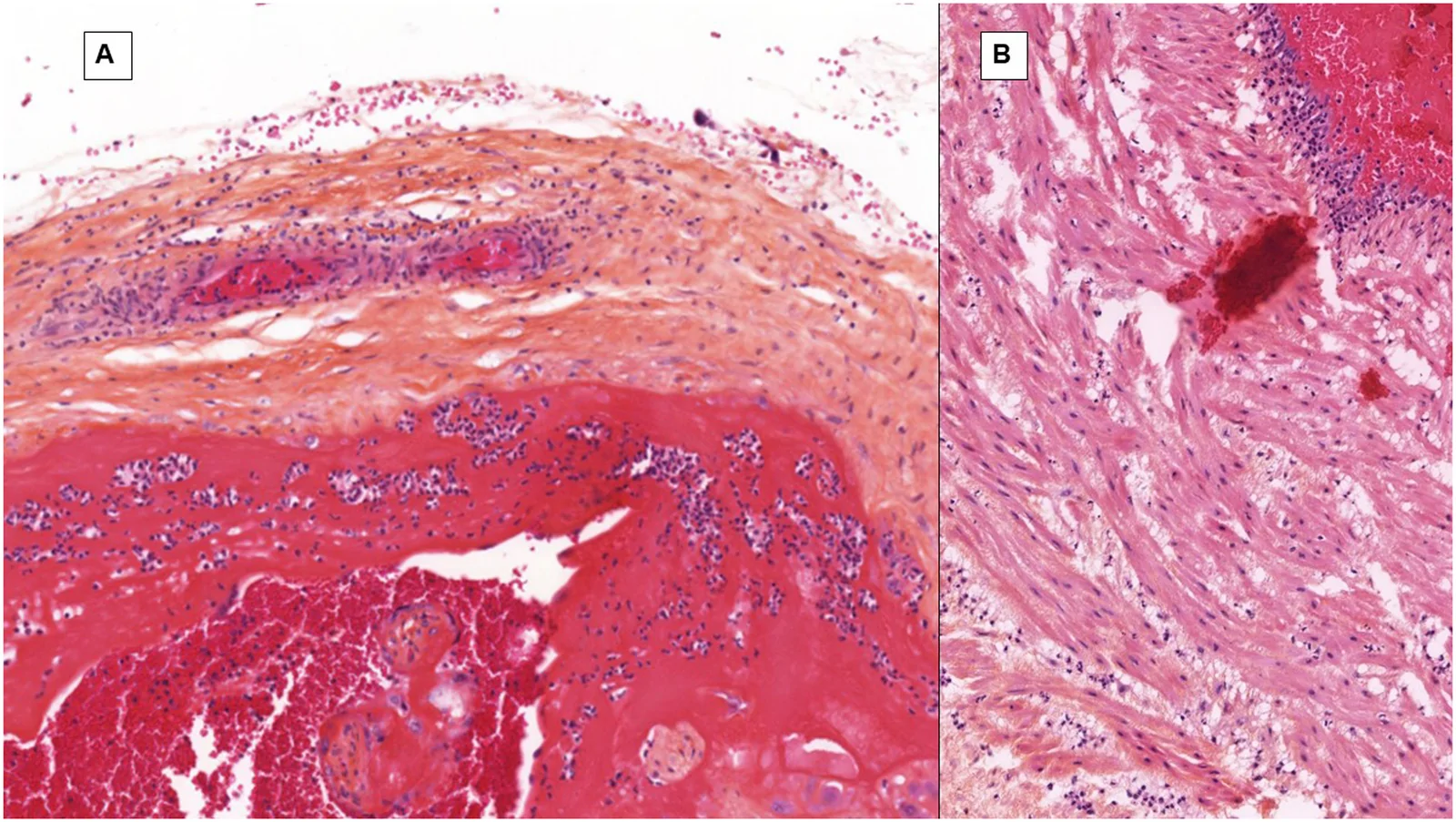
Histological aspects of an acute chorioamnionitis Histology demonstrating continuous bands of confluent polymorphonuclear neutrophils throughout the full thickness of the chorionic plate associated with **A**, chorionic vasculitis and **B**, umbilical phlebitis (hematoxylin-eosin-saffron staining × 10 and × 20).

Acute chorioamnionitis was the most prevalent histological lesion, affecting 174 cases (55.2%), predominantly showing only maternal inflammatory response stage 1 grade 1 (113/174 cases), equally in acute and nonacute groups without significant difference. Fetal inflammatory response was present in 49 cases (15.5%) (Table 1; Figure 2, A and B). Severe stage 2 maternal inflammatory lesions were observed in only 24 cases, and no stage 3 inflammatory lesion was identified.

Remoué. Omicron SARS-CoV-2 infection and placental outcomes. Am J Obstet Gynecol Glob Rep 2026.

**Figure 3:**
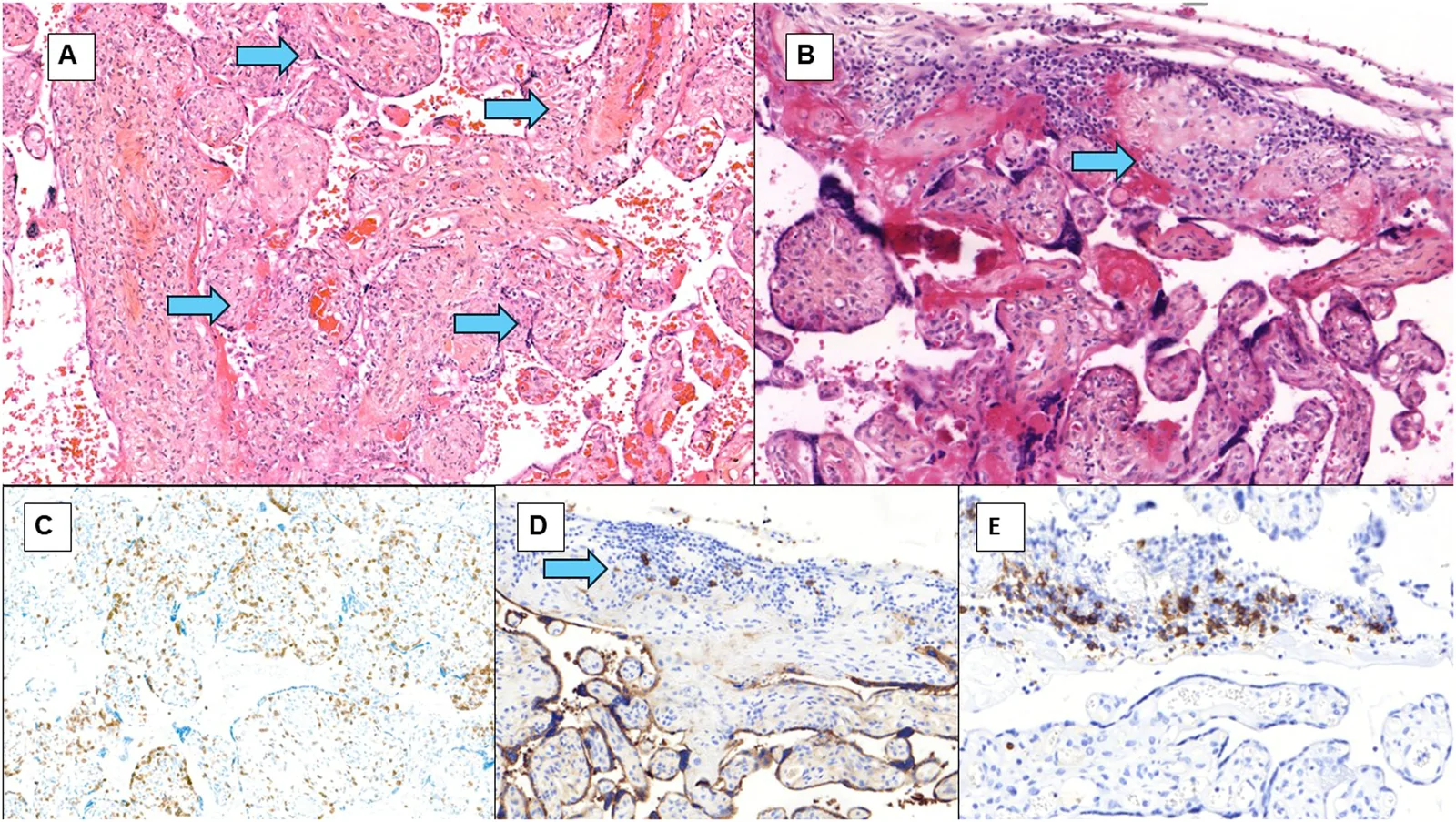
Histological and immunohistochemical features of high-grade chronic villitis and deciduitis **A**, High-grade distal chronic villitis (hematoxylin-eosin-saffron staining, × 20). B, Basal chronic villitis (hematoxylin-eosin-saffron staining, × 10). **C**, Positive immunohistochemical staining with anti-CD3 antibody (polyclonal, 1:100 dilution). **D**, Positive immunohistochemical staining with anti-CD138 antibody (monoclonal, MI15, M7228, 1:50 dilution,). **E**, Positive immunohistochemical staining with anti-CD20 antibody (clone L26, 1:100 dilution).

Chronic inflammation was observed in 65 cases (20.6%), predominantly chronic villitis in 47/315 (14.7%), with 11.4% being low-grade and 11/315 (3.4%) being high-grade (Table 1; Figure 3, A–E). Out of 12, 7 cases of chronic deciduitis were associated with parabasal chronic villitis (*P*<.001); 5 of them were high-grade chronic villitis (*P*<.001) (Figure 3, B–D). No cases of chronic chorioamnionitis or eosinophilic vasculitis were found.

Remoué. Omicron SARS-CoV-2 infection and placental outcomes. Am J Obstet Gynecol Glob Rep 2026.

**Figure 4:**
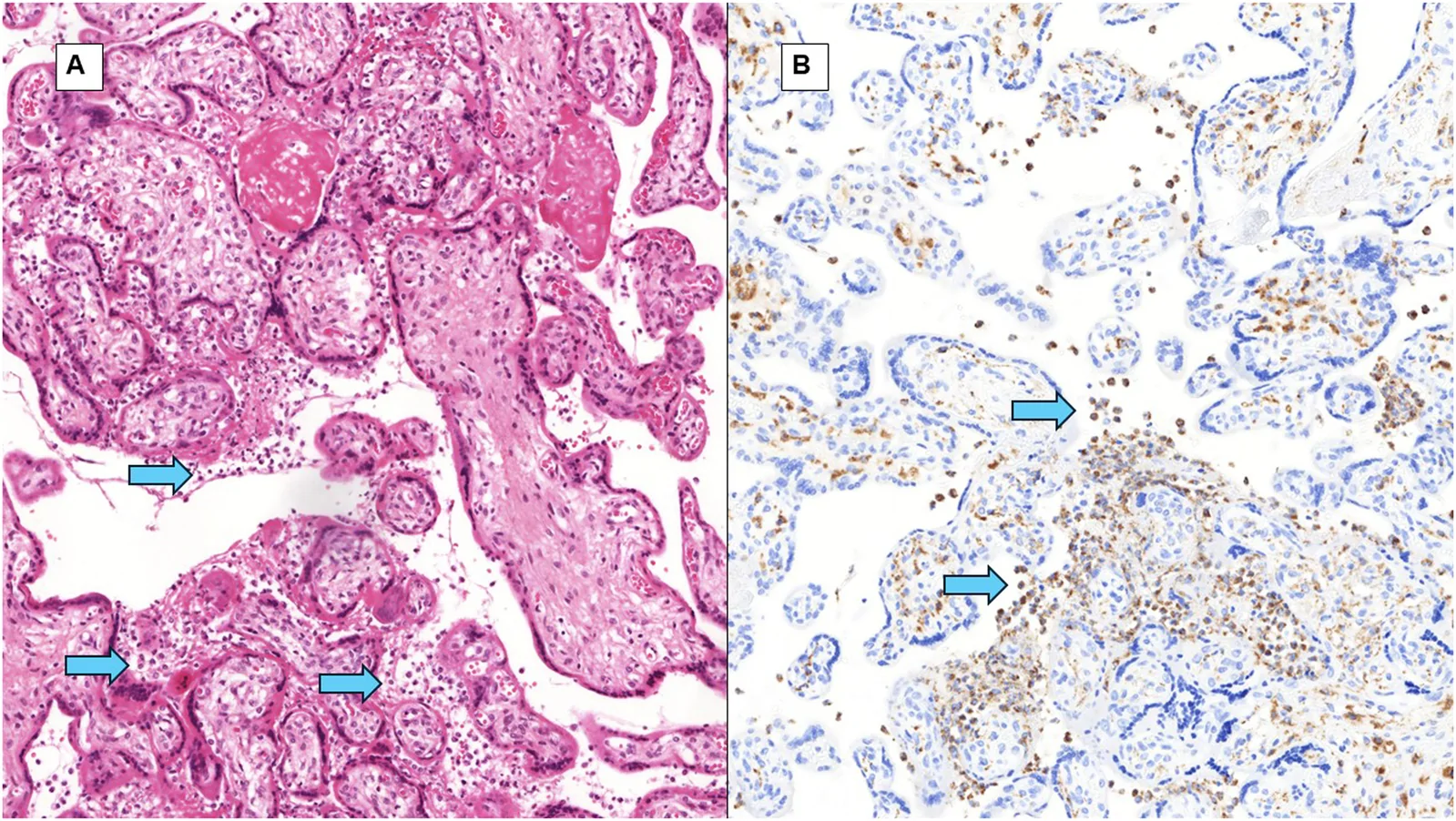
Histological and immunohistochemical features of chronic histiocytic intervillositis, showing intervillous macrophages **A**, hematoxylin-eosin-saffron staining, × 10. **B**, positive immunohistochemical staining with anti-CD68 antibody (clone PG-M1, 1:100 dilution)

CHI was found in 6 (1.9%) cases, all of which were low-grade (Figure 4, A and B), and all were associated with chronic villitis; the placentitis triad was not observed. All cases with chronic inflammation had negative SARS-CoV-2 immunohistochemistry.

Remoué. Omicron SARS-CoV-2 infection and placental outcomes. Am J Obstet Gynecol Glob Rep 2026.

**Figure 5:**
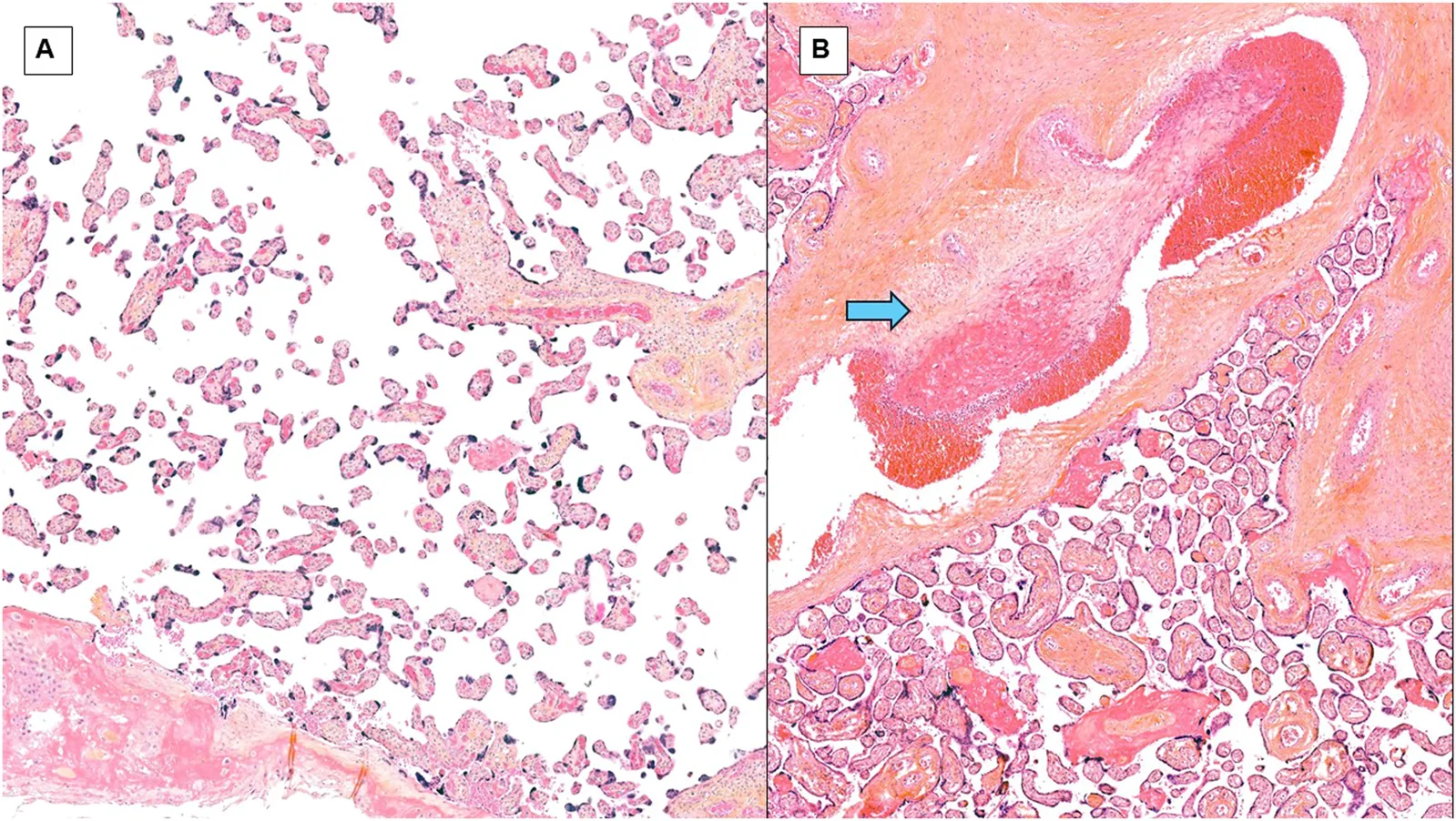
Histological features of maternal and fetal vascular malperfusion lesions **A**, Maternal vascular malperfusion lesions with reduced villous density, distal villous hypotrophy, and an increased number of syncytiotrophoblastic bridges (hematoxylin–eosin–saffron staining, × 5). **B**, Fetal vascular malperfusion lesion with thrombosis in a large troncular vessel (hematoxylin-eosin-saffron staining, × 10).

MVM features were observed in 49.2% of cases at similar rates in both the acute and nonacute groups (Table 1; Figure 5, A). FVM lesions were found in 7.6% of cases and were more frequent in the nonacute group compared with the acute group, without a significant difference (4.9% vs 9.2% [*P*=.19]) (Table 1; Figure 5, B).

Remoué. Omicron SARS-CoV-2 infection and placental outcomes. Am J Obstet Gynecol Glob Rep 2026.

### Comparison with published data from studies of individuals with non–Omicron variant SARS-CoV-2

Table 2 summarizes the comparison of the placental characteristics between our Omicron cohort and previous SARS-CoV-2 variants from 7 selected published studies, for a total of 1305 cases.^12^^,^^24^^,^^27^^,^^29^^,^^32^, ^33^, ^34^

**Table 2:** Placental pathologic findings: comparison between Omicron variant and published data from studies of individuals with non–Omicron variant SARS-CoV-2

Characteristics	Omicron group (n=327)	Sharp et al,^12^ 2020 (n=150)	Milot et al,^24^ 2023 (n=49)	Glynn et al,^27^ 2022 (n=90)	Patberg et al,^29^ 2021 (n=77)	Gulersen et al,^33^ 2020 (n=50)	Brien et al,^32^ 2021 (n=66)	Shanes et al,^34^ 2023 (n=823)	All variant groups	*P* value^a^
Macrsoscopic findings										
Placental weight <10th percentile, n (%)	13 (4.1)	NA	NA	NA	7 (9.1)	7 (14.0)	15 (22.7)	NA	29/193 (15.0)	.0001^a^
Infarct, n (%)	120 (38.0)	NA	18 (36.7)	NA	20 (26.0)	NA	3 (4.5)	NA	41/192 (21.4)	<.001^a^
Infarct >5%, n (%)	13 (4.1)	NA	NA	NA	NA	4 (8.0)	NA	NA	4/50 (8.0)	.26
Intervillous thrombosis, n (%)	71 (22.5)	NA	12 (24.5)	8 (10.0)	NA	13 (26.0)	NA	NA	33/189 (17.4)	.21
Micrscopic findings										
No placental lesion	61 (19.3)	NA	NA	NA	11 (14.3)	NA	10 (15.2)	NA	22/143 (15.4)	.35
MVM	155 (49.2)	69 (46.0)	44 (89.8)	27 (30.0)	NA	7 (14.0)	20 (30.3)	521 (63.3)	722/1228 (58.8)	<.010^a^
FVM, n (%)	24 (7.6)	53 (35.3)	22 (44.9)	16 (17.8)	25 (32.5)	4 (8.0)	18 (27.3)	51 (6.2)	189/1305 (14.5)	<.001^a^
ACA, n (%)	174 (55.2)	NA	11 (22.4)	10 (11.1)	19 (24.7)	NA	NA	NA	40/216 (18.5)	<.001^a^
ACA maternal response, n (%)	174 (55.2)	NA	NA	10 (11.1)	NA	NA	5 (7.6)	NA	15/156 (9.6)	<.001^a^
ACA fetal response, n (%)	49 (15.5)	NA	NA	6 (6.7)	10 (13.0)	NA	1/31 (3.2)	NA	17/198 (8.6)	.020^a^
VUE, n (%)	47 (14.7)	NA	8 (16.4)	19 (21.1)	16 (20.8)	2 (4.0)	3 (4.5)	NA	48/323 (14.9)	>.990
High grade, n (%)	11 (3.4)	NA	NA	6 (6.7)	NA	NA	NA	NA	6/90 (6.7)	.220
Low grade, n (%)	36 (11.4)	NA	NA	13 (14.4)	NA	NA	NA	NA	13/90 (14.4)	.460
CHI, n (%)	6 (1.9)	NA	0 (0)	NA	3 (3.9)	NA	NA	NA	3/77 (3.9)	.380
Chronic deciduitis, n (%)	12 (3.8)	NA	5 (10.2)	NA	NA	NA	7 (10.6)	NA	17/162 (10.5)	<.010

*ACA*, acute chorioamnionitis; *CHI*, chronic histiocytic intervillitis; *FVM*, fetal vascular malperfusion; *MVM*, maternal vascular malperfusion; *NA*, not available; *VUE*, villitis of unknown etiology.

a *P* value between Omicron group and all variant groups.

Macroscopically, 15% of placentas had a weight below the 10th percentile, vs 4.1% in placentas from pregnancies with maternal Omicron SARS-CoV-2 (*P*=.001); and 21.4% had at least 1 macroscopic infarct vs 38% in the Omicron cohort (*P*<.001).

Histologically, acute chorioamnionitis was more frequent in Omicron cohort placentas (55.2% vs 18.5% [*P*<.001]), notably in cases of fetal inflammatory response (15.5% vs 8.6% [*P*=.02]). Chronic villitis occurred at similar rates in the Omicron cohort and previou variant cohorts (14.7% vs 14.9%), but CHI and chronic deciduitis were more frequent in previousvariant cohorts: 3.9% in 1 study^29^ vs 1.9% in the Omicron cohort (*P*=.38), and 10.5% vs 3.8 %, respectively (*P*<.01). Vascular malperfusion lesions were significantly more frequent in previous-variant cohorts for MVM (58.8% vs 49.2% [*P*<.01]) and FVM (14.5% vs 7.6% [*P*<.001]).

### Comparison with published data of individuals without SARS-CoV2 from previous studies examining the association between SARS-CoV-2 infections and placental pathology

Table 3 summarizes the comparison of the placental characteristics between our Omicron cohort and SARS-CoV-2-negative controls from 8 published studies for a total of 2849 cases.^8^^,^^27^^,^^29^^,^^32^, ^33^, ^34^, ^35^, ^36^

**Table 3:** Placental pathologic findings: comparison between the Omicron variant and negative SARS-CoV-2 placentas from previous published studies

Characteristics	Omicron group (n=327)	Glynn et al,^27^ 2022 (n=188)	Wong et al,^8^ 2022 (n=47)	Patberg et al,^29^ 2021 (n=56)	Bertero et al,^35^ 2021 (n=58)	Brien et al,^32^ 2021 (n=49)	Gulersen et al,^33^ 2020 (n=50)	Shanes et al,^34^ 2023 (n=185)	Salafia et al,^36^ 2024 (n=2216)	All control groups	*P* value^a^
Macrsoscopic findings											
Placental weight <10th percentile, n (%)	13 (4.0)	NA	NA	7 (12.5)	NA	10 (20.4)	10 (20.0)	NA	NA	27/155 (17.4)	<.001^a^
Infarct, n (%)	120 (38.0)	NA	6 (12.8)	9 (16.1)	3 (5.2)	3 (6.1)	NA	NA	NA	21/210 (10.0)	<.001^a^
Intervillous thrombosis, n (%)	71 (22.5)	25 (14.4)	NA	NA	9 (15.5)		8 (16.0)	NA	NA	42/296 (14.2)	.010^a^
Micrscopic findings											
No placental lesion	61 (19.3)	NA	NA	27 (48.2)	NA	24 (49.0)	NA	NA	542 (24.5)	593/2321 (25.5)	.020^a^
MVM, n (%)	155 (49.2)	42 (24.1)	22 (46.8)	NA	NA	7 (14.3)	9 (18.0)	87 (47.0)	NA	167/519 (32.2)	<.001^a^
FVM, n (%)	24 (7.6)	23 (13.2)	0 (0)	2 (3.9)	NA	10 (20.4)	6 (12.0)	12 (6.5)	NA	53/575 (9.2)	.450
ACA, n (%)	174 (55.2)	40 (21.2)	6 (12.8)	11 (19.6)	7 (12.1)	NA	NA	NA	NA	64/349 (18.3)	<.001^a^
ACA maternal response, n (%)	174 (55.2)	25 (13.3)	NA	NA	NA	8 (16.3)	NA	NA	NA	33/237 (13.9)	<.001^a^
ACA fetal response, n (%)	49 (15.5)	13 (6.9)	NA	6 (10.7)	NA	7/38 (18.4)	NA	NA	NA	26/282 (9.2)	.020^a^
VUE, n (%)	47 (14.7)	24 (12.8)	2 (4.3)	4 (7.1)	0 (0)	1 (2.0)	1 (2.0)	NA	319 (14.4)	351/2664 (13.2)	.380
High grade, n (%)	11 (3.4)	11 (6.3)	1 (2.1)	NA	NA	NA	NA	NA	NA	12/235 (5.1)	.390
Low grade, n (%)	36 (11.4)	13 (7.5)	1 (2.1)	NA	NA	NA	NA	NA	NA	14/235 (6.0)	.030^a^
CHI, n (%)	6 (1.9)	NA	2 (4.3)	0 (0)	0 (0)	NA	NA	NA	NA	2/161 (1.2)	.720
Chronic deciduitis, n (%)	12 (3.8)	NA	1 (2.1)	NA	0 (0)	1 (2.0)	NA	NA	94 (4.2)	96/2370 (4.1)	>.990

*ACA,* acute chorioamnionitis*; CHI*, chronic histiocytic intervillitis; *FVM*, fetal vascular malperfusion; *MVM*, maternal vascular malperfusion; *NA,* not available; *VUE*, villitis of unknown etiology*.*

a *P* value between Omicron group and all control groups.

Macroscopically, 17.4% of placentas had a weight below the 10th percentile vs 4.1% in the Omicron cohort placentas (*P*<.001), and 10% had at least 1 macroscopic infarct vs 38% (*P*<.001).

Acute chorioamnionitis was significantly more frequent in the Omicron cohort (55.2% vs 18.3% [*P*<.001]), notably in cases of fetal inflammatory response (15.5% vs 9.2% [*P*=.025]), and MVM (49.2% vs 32.2% [*P*<.001]). There was no significant difference concerning chronic villitis (14.7% vs 13.2% [*P*=.38]), CHI (1.2% vs 1.9% [*P*=.72]), chronic deciduitis (3.8% vs 4.1% [*P*>.99]), and FVM (7.6% vs 9.2% [*P*=.45]).

## Discussion

This retrospective cohort study included 315 placentas from pregnancies with diagnosed maternal Omicron variant SARS-CoV-2 infection. The viral infection mainly occurred in the third trimester and did not lead to a higher prevalence of preterm deliveries, preeclampsia, or FGR. The maternal and fetal outcomes were favorable, with only 1 stillbirth reported, which occurred 6 weeks after the infection and without placental histopathological anomalies.

Omicron cohort placentas were mostly eutrophic and did not show any SARS-CoV-2 placentitis.^15^, ^16^, ^17^, ^18^, ^19^, ^20^, ^21^, ^22^, ^23^ There was no significant difference between acute and nonacute groups, but we observed that chronic villitis was 2 times more frequent in the nonacute group. CHI was observed in 1.9% of Omicron cohort placentas, but all cases were associated with chronic villitis. We did not observe a higher rate of FVM in the acute group, as described by Glynn et al.^27^ In contrast, we observed more cases in the nonacute group without significant difference.

Comparison with a control cohort and cohorts infected with previous COVID-19 variants demonstrated a significantly lower rate of hypotrophic placentas in our cohort. The incidences of placental hypotrophy, macroscopic infarctions, and histological MVM lesions were not correlated among the different cohorts, contrary to what would be expected, with no clear scientific explanation.

Moreover, we observed a higher incidence of acute histological chorioamnionitis. This inflammation is usually triggered by microbial infection of the amniotic fluid, which ascends through the intrauterine route.^38^^,^^39^ Although most of the cases exhibited only stage 1, grade 1 maternal inflammatory response according to the Amsterdam Consensus criteria,^31^ which some authors regard as part of the normal physiological process of labor,^40^ a fetal inflammatory response was identified in 15.5% of cases, without being associated with preterm delivery.^38^ Chronic inflammation was found in 16.2% of Omicron cohort placentas, mostly chronic villitis, at a rate comparable with the published data,^12^^,^^24^^,^^27^^,^^29^^,^^32^, ^33^, ^34^ and mostly with a parabasal pattern associated with chronic deciduitis.^41^^,^^42^

Of note, three histologic findings, including chronic deciduitis and maternal and fetal vascular malperfusion lesions, were significantly higher in the previous-variant cohorts. Shanes et al^34^ reported MVM features in 65% of placentas during the α and γ era, 82% in the δ era, and 55% in the Omicron cases. This point is in accordance with the relatively good fetomaternal prognosis of maternal Omicron infection, as vascular malperfusion lesions are associated with complications such as FGR, preeclampsia, or preterm delivery. Several studies failed to demonstrate a parallel increase in clinical manifestations of placental insufficiency, including preeclampsia and FGR, and described this apparent clinicopathological dissociation,^12^^,^^24^^,^^27^^,^^29^^,^^34^ suggesting that many vascular lesions remain subclinical or are insufficiently extensive to impair placental function.

Moreover, it is noteworthy that the percentage of Omicron cohort placentas without macroscopic or histopathological lesions (18.7%) in our study closely matched the figure observed in the at-term control placental cohort of Romero et al^40^ (16.6%), even though, in comparison with the control cohort, we observed significantly more intervillous thrombosis and MVM lesions. Therefore, the relatively high prevalence of macroscopic infarctions observed in the Omicron cohort remains difficult to explain. A possible explanation is interobserver variability in pathological assessment, with a lower threshold for recording even small focal infarctions as macroscopic infarcts.

The retrospective nature of the study introduced potential biases. The 2 senior pathologists who analyzed the placentas were aware of the viral status of the mothers. An important limitation of this study is that the comparator groups were derived from previously published studies rather than from contemporaneously recruited participants within our study. Although these cohorts were broadly comparable with respect to maternal and obstetrical characteristics, we acknowledge the limitations inherent to historical comparisons, including differences in ethnic composition and incomplete reporting of obstetrical history. Regarding the comparison with the previously published studies, the timing of maternal infection was not strictly comparable, with a predominance of third-trimester infections in the Omicron cohort. This heterogeneity is acknowledged as a limitation of our study and may partly explain differences in placental findings between the cohorts.

However, the present study has some strengths. This study presents a placental cohort of more than 300 cases specifically focused on Omicron SARS-CoV-2 variant, with placental findings evaluated according to the infecting variant. The study design was multicentric in terms of recruitment, covering 6 maternities in Western Brittany. All positive cases of SARS-CoV-2 were included, even in cases of uneventful deliveries. A standardized protocol for sampling and histological analysis was used, in accordance with the Amsterdam consensus recommendations, and the placental analysis was performed by 2 senior specialized pathologists.

## Conclusion

This large retrospective cohort study of Omicron-variant placentas suggests favorable pregnancy outcomes for mothers and fetuses with no increased clinical complications. We showed a significantly lower rate of hypotrophic placentas below the 10th percentile, chronic deciduitis, and MVM and FVM lesions in the Omicron cohort vs the previous-variant cohorts. We did not observe an increased incidence of chronic inflammation or the placentitis triad in comparison with the control cohort. There was no significant difference between the acute (delivery occurring less than 14 days after the infection) and nonacute groups. Our findings indicate that Omicron variant infection is linked to different placental histopathological alterations than previous variants, underscoring the importance of continued placental surveillance and further studies to clarify the maternal and fetal impact of each viral variant and improve knowledge of the protective barrier of the placenta.

## CRediT authorship contribution statement

**Annabelle Remoué:** Writing – review & editing, Writing – original draft, Visualization, Validation, Supervision, Software, Methodology, Investigation, Formal analysis, Data curation, Conceptualization. **Yurina Suazo:** Writing – original draft, Software, Methodology, Investigation, Data curation, Conceptualization. **Claire de Moreuil:** Writing – original draft, Validation, Supervision, Methodology, Investigation. **Karine Morcel:** Investigation, Data curation. **Christophe Tremouilhac:** Investigation, Data curation. **Camille Sauvée:** Methodology, Data curation. **Arnaud Uguen:** Writing – original draft, Validation, Software, Methodology, Conceptualization. **Pascale Marcorelles:** Writing – review & editing, Writing – original draft, Validation, Supervision, Methodology, Investigation, Data curation, Conceptualization.
